# Nondestructive Analysis of Internal Quality in Pears with a Self-Made Near-Infrared Spectrum Detector Combined with Multivariate Data Processing

**DOI:** 10.3390/foods10061315

**Published:** 2021-06-07

**Authors:** Xin Wu, Guanglin Li, Fengyun He

**Affiliations:** 1 Department of Agricultural Engineering, College of Engineering and Technology, Southwest University, Chongqing 400715, China; wuxin@cqcet.edu.cn (X.W.); hefengyun@email.swu.edu.cn (F.H.); 2 Department of Electronics and Internet of Things, Chongqing College of Electronic Engineering, Chongqing 401331, China

**Keywords:** variable selection methods, variable stability and cluster analysis algorithm (VSCAA), internal quality, near-infrared (NIR) spectroscopy, pears

## Abstract

The consumption of pears has increased, thanks not only to their delicious and juicy flavor, but also their rich nutritional value. Traditional methods of detecting internal qualities (e.g., soluble solid content (SSC), titratable acidity (TA), and taste index (TI)) of pears are reliable, but they are destructive, time-consuming, and polluting. It is necessary to detect internal qualities of pears rapidly and nondestructively by using near-infrared (NIR) spectroscopy. In this study, we used a self-made NIR spectrum detector with an improved variable selection algorithm, named the variable stability and cluster analysis algorithm (VSCAA), to establish a partial least squares regression (PLSR) model to detect SSC content in snow pears. VSCAA is a variable selection method based on the combination of variable stability and cluster analysis to select the infrared spectrum variables. To reflect the advantages of VSCAA, we compared the classical variable selection methods (synergy interval partial least squares (SiPLS), genetic algorithm (GA), successive projections algorithm (SPA), and bootstrapping soft shrinkage (BOSS)) to extract useful wavelengths. The PLSR model, based on the useful variables selected by SiPLS-VSCAA, was optimal for measuring SSC in pears, and the correlation coefficient of calibration (Rc), root mean square error of cross validation (RMSECV), correlation coefficient of prediction (Rp), root mean square error of prediction (RMSEP), and residual predictive deviation (RPD) were 0.942, 0.198%, 0.936, 0.222%, and 2.857, respectively. Then, we applied these variable selection methods to select the characteristic wavelengths for measuring the TA content and TI value in snow pears. The prediction PLSR models, based on the variables selected by GA-BOSS to measure TA and that by GA-VSCAA to detect TI, were the best models, and the Rc, RMSECV, Rp and RPD were 0.931, 0.124%, 0.912, 0.151%, and 2.434 and 0.968, 0.080%, 0.968, 0.089%, and 3.775, respectively. The results showed that the self-made NIR-spectrum detector based on a portable NIR spectrometer with multivariate data processing was a good tool for rapid and nondestructive analysis of internal quality in pears.

## 1. Introduction

Pears are one of the most popular fruits in the world. Pears are typically used as food; not only are they sweet, juicy, and delicious, with some acidity, but they are also rich in nutrition and contain a variety of vitamins and cellulose. The tastes and textures of different kinds of pears are different. More than 60% of the world’s pears are produced in China [1]. Consumers pay attention to the external quality of pears, including size, color, and shape, as well as to the internal quality of pears, including the sugar content, acidity, and taste. After harvest, the detection and grading of the fruit’s internal quality always plays an important role in its commercialization [2]. The soluble solid content (SSC) not only affects the internal quality and price of fresh fruit, but also determines the fruit maturity and harvest time [2]. The titratable acidity (TA) is often used to estimate the ripening time of pears: as fruits get closer to ripening time, their acidity decreases and their taste tends to be sweeter. The taste index (TI) is defined as the ratio of SSC to TA. This index can be used to determine the taste and ripening stage of pears. Traditional methods of detecting internal qualities of fruit are reliable, but they are destructive, time-consuming, and polluting. Thus, it is impossible for these traditional chemical measurement methods to detect internal quality of fruit rapidly and nondestructively. Therefore, the conventional physicochemical analysis methods currently used to evaluate the internal quality of fruit do not meet consumers’ requirements for fruit with consistency and high quality.

Over the past few decades, various studies have been conducted using near-infrared (NIR) or visible-NIR (vis-NIR) spectroscopy as rapid and nondestructive methods for determination of the internal quality in fresh fruit. Li et al. [3] used the vis-NIR spectroscopy spectrometric technique to measure the SSC and firmness of pear fruit in the wavelength range of 400–1800 nm. The prediction results showed that the correlation coefficient of prediction (Rp), root mean square error of prediction (RMSEP), and residual predictive deviation (RPD) were 0.9486, 0.3244%, and 3.1598 for SCC and 0.8955, 1.1077%, and 2.2469 for firmness. These results showed that vis-NIR spectroscopy could be applied as a fast and accurate alternative method for the nondestructive determination of SSC and firmness of pears. The research team first used the long-wave infrared hyperspectral imaging in the wavelength of 1000–2500 nm to measure the SSC in pears [4]. Results of the Monte Carlo-uninformative variable elimination-successive projections algorithm-partial least square (MC-UVE-SPA-PLS) model using 18 selected characteristic variables were an Rp of 0.88 and RMSEP of 0.35. The team also used the NIR and portable vis-NIR spectroscopy for nondestructive determination of SSC in pears, combined with an informative variable selection algorithm, and two calibration algorithms, including linear regressions of multiple linear regression (MLR) and nonlinear regression of least-square support vector machine (LS-SVM), such as MC-UVE-SPA-MLR [5] and MC-UVE-SPA-LS-SVM [6], were used for measurement. They also established multicultivar models for the determination of SSC in pears [2] and conducted a comparative study for the quantitative determination of SSC, pH, and firmness of pears by vis-NIR spectroscopy [7]. Tian et al. [8] developed a fruit surface feature classification and multivariate regression analysis for the nondestructive prediction of SSC in pears, based on the vis-NIR transmission spectra of “Korla” pears, with a portable spectrometer instrument; the Rp and RMSEP were 0.9368 and 0.5256%, respectively. Lee and Han [9] nondestructively detected the sugar content of Korean pears using NIR diffuse-reflectance spectroscopy, and the prediction accuracy was evaluated to be about 0.24%. Wang et al. [10] used the vis-NIR spectroscopy combined with chemometric methods for the nondestructive detection of the juiciness of pears, and the external verification determination coefficient (Rv) was 0.93, and the root mean square error of cross validation (RMSECV) was 0.97%. Yu et al. [11] used optical properties and diffuse reflectance in the 900–1700 nm spectral region for prediction and comparison of models for SSC determination in “Ya” pears. Xu et al. [12] developed an application for the online determination of sugar content in pears based on variable selection in vis- and NIR spectra, with Rv = 0.880 and RMSEP = 0.459% for the validation set. Rittiron et al. [1] used NIR spectroscopy in the short wavelength region (700–1100 nm) for rapid and nondestructive detection of water-core and sugar content in Asian pears for commercial trade. Travers et al. [13] predicted and compared preharvest pear dry matter (DM) and SSC, based on two near-spectral ranges (680–1000 nm and 1100–2350 nm). Models based on longer NIR spectra were more successful for both parameters (DM/SSC: Rv = 0.78–0.84; RMSECV = 0.78/0.44; latent variables (LVs) = 6/7). Adebayo et al. [14] used absorption and reduced scattering coefficients based on the vis- and shortwave (SW)-NIR wavelength range for nondestructive analysis of fruit flesh firmness and SSC in pears. Nicolaï et al. [15] used time-resolved and continuous wave NIR reflectance spectroscopy to predict the SSC and firmness of pears. Sun et al. [16] used online vis- and NIR spectroscopy for simultaneous measurement of brown core and SSC in pears. Wang et al. [17] developed multicultivar models to predict the SSC and firmness of European pears (*Pyrus communis* L.) using portable vis-NIR spectroscopy. Yu et al. [18] developed a deep learning method for predicting firmness and SSC of postharvest Korla fragrant pears using vis-NIR hyperspectral reflectance imaging. Choi et al. [19] used a portable, nondestructive tester, integrating vis-NIR reflectance spectroscopy, to detect the sugar content in Asian pears. Passos et al. [20] nondestructively detected soluble solids content for “Rocha” pears based on vis-SWNIR spectroscopy under real-world sorting facility conditions. Liu et al. [21] used NIR diffuse reflectance spectroscopy combined with variable selection algorithms for optimized prediction of sugar content in snow pears. Sheng et al. [22] nondestructively measured lignin content in Korla fragrant pears, based on NIR spectroscopy, and the Rp, RMSEP, and RPD were 0.87, 1.36%, and 2.03, respectively. Fan et al. [23], Zhang et al. [24], Lee et al. [25], and Li et al. [19,26] used vis-NIR and NIR hyperspectral imaging technology for fast and nondestructive prediction of SSC and firmness, based on the competitive adaptive reweighted sampling-successive projections algorithm-partial least square (CARS-SPA-PLS) models, and the Rp and RMSEP of the prediction sets were 0.876 and 0.491% for SSC and 0.867 and 0.721% for firmness. They also used this technology for the prediction of internal sugar content in Dangshan pears, and the Rp and RMSEP of CARS-PLS were 0.8971 and 0.3937%, and they were 0.8969 and 0.3482% for (genetic algorithm: GA) GA-SPA-PLS. They used the technology to detect the physical damage of pears as well as for the identification of the type of wax on pears, with an identification accuracy of 99.07% for calibration and 95.83% for the prediction sets. They used the technology (400–1000 nm) for nondestructive variety discrimination and prediction of SSC and firmness of pears, with a correlation coefficient (r) of 0.9977 for firmness and 0.9924 for SSC.

Qualitative and quantitative analysis techniques of NIR spectroscopy have been widely applied to rapidly and nondestructively detect external and internal quality of fruit and vegetables because of their characteristics of fast response speed, multiple analysis components, and accurate prediction [27,28]. Modern spectroscopy instrumentation is composed mainly of optical and electronic components, however, which are easily affected by the surrounding environment. As a result, thousands of spectral data obtained by spectrometers inevitably contain useless interference information. The thousands of spectral data are too complicated to establish a calibration and prediction model, and the calibration process is quite time-consuming, which is even worse than the prediction performance of the model. Therefore, variable selection is an essential step before establishing the calibration and prediction models [29,30,31,32].

The partial least square regression (PLSR) model has been widely established for its calibration and prediction analysis in spectroscopy technology because of its simple operation and high prediction accuracy. Variable selection is an important step in the multivariate model, because the predictive ability of the model can be increased, and the complexity of the model can be reduced [33]. The regression coefficient β is an important index parameter in the PLSR model, and several classical variable selection algorithms based on this regression coefficient include Monte Carlo non-information variable elimination (MCUVE), competitive adaptive reweighted sampling (CARS), and bootstrapping soft shrinkage (BOSS). Useful variables are selected according to the stability of each variable in the MCUVE methods [29,32,33,34]. A large number of sample spaces can be generated using MCUVE, and PLSR models are built for each sample space. The stability of each variable can be obtained according to the mean value and standard deviation of PLSR regression coefficient. When the stability of a variable is less than the threshold, the variable is considered to be useless and is eliminated. The variables that are selected are still large in the MCUVE application, however, and the value of threshold has a significant impact on the results of variable selection. In the CARS algorithm, variables can be selected according to the absolute value of the regression coefficient [10,29,31,32,34,35,36]. The variables with smaller absolute values of the regression coefficient must be removed. The absolute value of the regression coefficient will change, however, with the change of the sample space, which can result in the eliminated variables containing useful variables. The BOSS algorithm [29,32] is a kind of spectral line selection method based on variable space. Submodels are established based on a large number of variable spaces generated by the weighted bootstrap sampling (WBS) method and the Monte Carlo algorithm. PLSR models are built based on the submodel, and then, the absolute value of the regression coefficient is calculated and normalized to update the weight of each variable. The variables with bigger values of weight have a greater chance of being selected in the next iteration. The BOSS algorithm, however, considers only a characteristic factor “regression coefficient” in the variables space. Therefore, the selected variables may not be optimal. In light of the variable selection method problems, we proposed a variable selection method based on the combination of variable stability and cluster analysis algorithm (VSCAA) to select the infrared spectrum variables in this work.

In summary, development of a nondestructive method combined with a variable selection algorithm for the detection of the SSC, TA, and TI properties of pears is necessary. Therefore, the objectives of this study were as follows: (1) set up an ultra-compact NIR spectroscopy system to collect the spectral data; (2) preprocess the raw spectra by different preprocessing methods to eliminate the light-scattering effects; (3) utilize and compare SiPLS, SPA, BOSS, GA, and VSCAA methods as well as their combination to select the feature variables and enhance the model’s prediction ability; and (4) evaluate the performance of the PLS model based on the independent verification datasets.

## 2. Materials and Methods

### 2.1. Sample Collection

**Figure 1:**
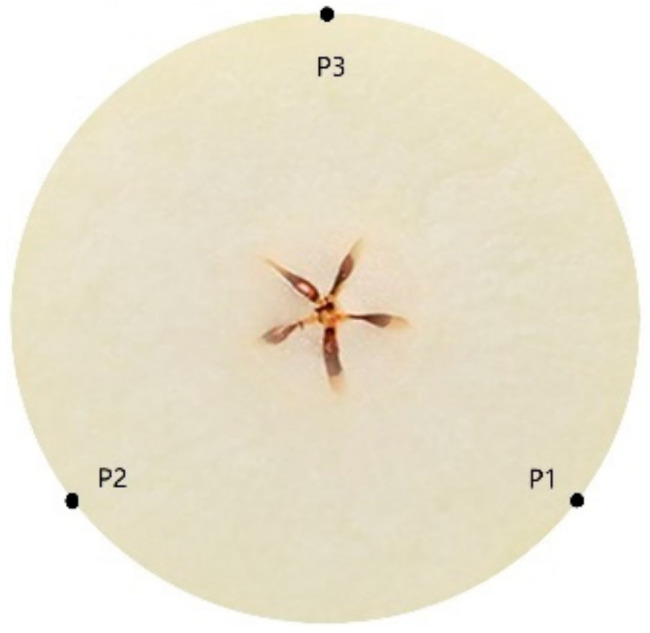
The diagram of the three measuring points (P1, P2, and P3), which were marked around the equator of pear and set as a 120° angular distance.

We conducted this experiment in the summer of 2020 in a Spectral Analysis Laboratory at Southwest University, Chongqing, China. We purchased a total of 195 snow pears, a representative China pear, from a local fruit market for this research. To ensure the accuracy of the experiment, pears are washed, numbered, and then stored in an ice box (4 °C, 75% relative humidity (RH)) for 24 h to avoid moisture loss before the experiment was carried out [37]. Figure 1 shows that three measuring points (P1, P2, and P3) around the equator of the pear were set as a 120° angular distance.

### 2.2. Spectral Acquisition

**Figure 2:**
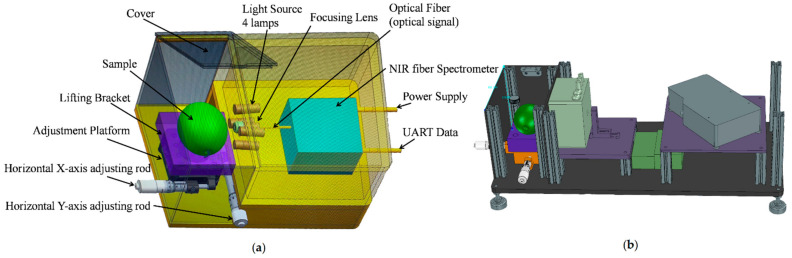
The near-infrared (NIR) spectroscopy system for spectral measurement. (**a**) Structure diagram of detector. (**b**) Design drawing of detector.

We established an ultra-compact NIR spectroscopy system for spectral measurement, as shown Figure 2. The system mainly consisted of a light source, NIR spectrometer, two-dimensional adjustment platform, and lifting bracket. The Vivo Light Source (Ocean Insight, Largo, FL, USA) consisted of four 5-watt tungsten halogen lamps and was adjusted at an angle of approximately 45° to verify a unit’s lighting, which is often applied to measure the diffuse reflection spectral of a sample. In this study, we used an NIR spectrometer (NIRQuest512-2.5, Ocean Insight) with a range of 900–2500 nm and a resolution of 6.3 nm for the core part of the detection system to collect spectra. A coated quartz focusing lens (74-UV, Ocean Insight) with a diameter of 5 mm and focal length of 10 mm was coupled to the front of the spectrometer to concentrate the light toward the entrance of the spectrometer and achieve a larger photosensitive range. We used a self-designed lifting bracket with a two-dimensional adjustment platform and a bracket to adjust the sample location in the optical sensing system to avoid ambient light interference. The spectral acquisition system can achieve the acquisition of NIR spectra from 900 to 2500 nm. In order to make the focusing lens closer to the sample surface, the pear was put onto the sample tray, and the vertical and horizontal positions of the bracket were adjusted. The integration time of the NIR spectrometer was set to 0.01 s and the average number of scan times was 5. We repeated this operation three times, which was accompanied by rotating the sample 120°. Therefore, each sample spectrum was measured three times for averaging.

### 2.3. Reference Value of SSC, TA, and TI

#### 2.3.1. Reference Measurements of SSC

We used a fully automatic desktop digital refractometer (RX-5000i-plus, ATAGO, Japan) to measure the SSC reference value of pears immediately after spectra collection [38,39,40]. The measurement range of the refractometer was 0–100% Brix, and the accuracy was ± 0.01%. When measuring the SSC value, pear juice was squeezed out of each sample and dripped onto the mirror of the refractometer using a pipette. We then detected and recorded the SSC value. Three SSC values were tested to obtain the average value at each marked point of each sample (Table 1).

#### 2.3.2. Reference Measurements of TA

We determined the TA value using a traditional titration method according to 942.15, described by the Association of Official Analytical Chemists (AOAC) [41]. We diluted 10 g of pear fruit in 50 mL deionized water and added 0.5 mL phenolphthalein (10 g/L, *w*/*v*), which was used as an indicator. We used 0.1 mol/L NaOH solution (standardized) to neutralize the acidity and stopped the titration when a pink color persisted for 30 s. We then measured and recorded the TA value. We used the average value for three measurements at each marked point in the latter analysis (Table 1).

#### 2.3.3. Reference Measurements of TI

We defined TI as the ratio of SSC to TA; see Equation (1):

(1) $$V_{\mathrm{TI}}=\frac{V_{\mathrm{SSC}}}{V_{\mathrm{TA}}}$$

where V_TI_ is the value of TI, V_ssc_ is the value of SCC, and V_TA_ is the value of TA. For the convenience of calculation, we made a global numerical transformation of VTI; see Equation (2):

(2) $${\mathrm{nV}}_{\mathrm{TI}}=V_{\mathrm{TA}}\times P_{TA}+V_{TI}\times 0.119\times P_{TI}$$

where nV_TI_ is new value of V_TI_, and this value participated in the modeling in the later data processing. P_TA_ is the percentage of V_TA_ in the range of 0–100%, and P_TI_ is the percentage of V_TI_ in the range of 0–100%, which ensures that the sum of P_TA_ and P_TI_ is 100%. In this work, the optimal PLSR model had the best performance base on the nV_TI_ by setting a P_TA_ of 90% and P_TI_ of 10%, with a (Rc) of 0.962, RMSEC of 0.084%, Rp of 0.984, RMSEP of 0.063%, and RPD of 5.14, respectively. Therefore, the latter spectral preprocessing and variable selection are based on the following Equation (3):

(3) $${\mathrm{nV}}_{TI}=V_{TA}\times 90\%+V_{TI}\times 0.119\times 10\%$$

**Figure 3:**
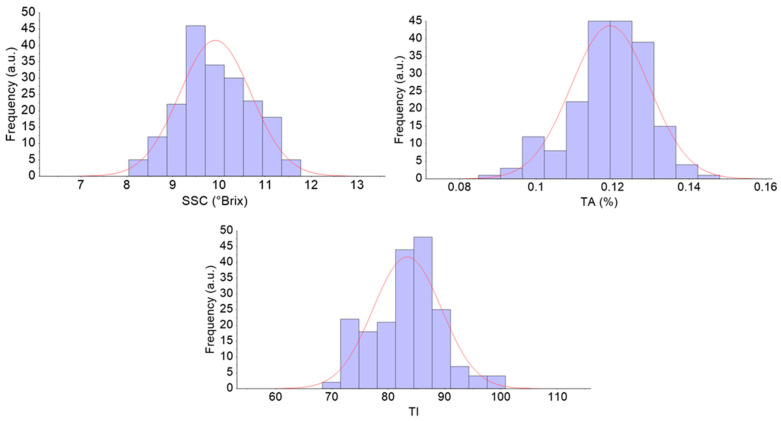
The SSC content distribution of all pears. a.u: arbitrary unit. SSC: soluble solid content; TA: titratable acidity; TI: taste index.

The SSC, TA, and TI of all 195 pears were 8.04–11.78°Brix, 0.0851–0.1480%, and 68.3–100.8, respectively. As shown in Figure 3, the concentration of the sample was in accordance with the normal distribution. The SSC distribution of the calibration set and the prediction set are given in Table 1.

### 2.4. Spectrum Pre-Processing

The main spectra preprocessing methods [10] include baseline correction, scattering correction, smoothing processing, and scale scaling. Baseline correction is used to deduct the influence of instrument background or drift on signal, including first derivative (D1) and second derivative (D2). Scattering correction is used to eliminate the influence of scattering caused by uneven particle distribution and different particle size on the spectrum, including multiplicative scatter correction (MSC) and standard normal variate (SNV) transformation. Smoothing processing is used to eliminate the random noise in the spectrum signal and improve the signal-to-noise ratio of the sample signal, including moving average smoothing (MAS) and Savitzky–Golay (SG) smoothing. Scale scaling is used to eliminate the adverse effects caused by too large scale differences of data, including centralization, Pareto scaling, and maximum and minimum normalization. In this work, we used MAS (smoothing points = 11), normalization, MSC, SNV, D1, and D2 (polynomial order = 2, smoothing points = 11) methods to pretreat the raw NIR spectra using the software Unscrambler X v. 10.4 (Camo Analytics, Oslo, Norway).

### 2.5. Description of VSCAA

We divided the variables into sample space and variable space and calculated the stability of each variable in the sample space and the frequency of each variable in the variable space. We used the WBS to divide the variables into useful variables and useless variables, according to the stability of each variable, and applied the exponential decay function to eliminate the useless variables with low frequency in the variable space. To realize the selection of variables, this method was named VSCAA.

#### 2.5.1. Definition of Variable Stability in the Sample Space

We assumed that the NIR spectra data matrix of sample is X_n×p_, the number of samples is n, and the number of spectral lines (variable) is *p* in the matrix X, and that n << *p*; y is the concentration information matrix of the analysis sample. When the components contained in the sample are 1, y is the concentration information vector, and e is random error. The relationship between the spectral matrix and concentration information during the establishment of PLS regression model can be expressed as follows:

(4) y=Xβ+e

where β is the vector of the regression coefficient, ${\beta =[\beta }_{1}{,\beta }_{2},\cdots {,\beta }_{p}{]}^{T}$. The n1 samples were selected randomly from n samples as the sample space using the Monte Carlo algorithm. We repeated the operation M times and got the M sample spaces. The matrix of the regression coefficient **β**_p×M_ was obtained after the PLS regression model was established for each sample space; see Equation (4):

(5) $$S_{i}=\left|\frac{{\mathrm{mean}(\beta }_{i1}{,\beta }_{i2}\cdots \beta_{\mathrm{iM}})}{{\mathrm{std}(\beta }_{i1}{,\beta }_{i2}\cdots \beta_{\mathrm{iM}})}\right|$$

where mean (…) is the mean value of variables’ regression coefficient, std (…) is the standard deviation of each variable’s regression coefficient, and Si is the stability of the i-th variable.

#### 2.5.2. Variable Selection Method: VSCAA

When selecting characteristic variables, the VSCAA algorithm should consider not only the stability of each variable’s regression coefficient, but also the frequency of variables. When the stability of a variable is smaller, it has a higher probability of being considered a useless variable. At this time, if the frequency of the variable is also smaller, the variable will be forced to be eliminated. When the loop reaches the set number of times, the iteration will stop.

The specific implementation steps of the VSCAA algorithm were as follows:

##### Step 1

At the beginning of the iteration, the initial number of the variable was equal to the number of the wavelengths of the sample, and the number was *p*. The n1 samples were selected randomly from the n samples as the sample space, and the stability of each sample Si was calculated. The *p* samples were divided into useful variable and useless variable according to the value of Si, by the weighted bootstrap sampling method. Note that the number of useful variables was about 0.632 times the length of the variable.

##### Step 2

The number of samples n was constant. The Monte Carlo algorithm was applied to randomly select P1 variables from *p* variables. We repeated the previous operation W times and got W variable spaces. The PLS regression model was established for each variable space and the RMSE was obtained for each PLSR model. The αW models with smaller RMSE values were chosen from the W models, and the frequency of each variable fi was obtained.

##### Step 3

The exponential decay function was used to determine the remaining variables after each iteration. The formula of the exponential decay function is as follows:

(6) $$r_{i}{=\alpha e}^{-\mathrm{ki}}$$

where r_i_ is the number of the remaining variables,

(7) $$\alpha =(\frac{p}{2}{)}^{\frac{1}{N-1}}$$

(8) $$k=\ln(\frac{p}{2}{)}^{\frac{1}{N-1}}/(N-1)$$

where i is the i-th loop, and N is the number of loops. When the number of remaining variables was greater than the number of useful variables in step 1, then the variables with low frequency in step 2 were removed from the useless variables. When the number of remaining variables was less than the number of useful variables, then all of the useless variables should have been removed and the variables with low stability would be eliminated from the useful variables.

##### Step 4

The PLS regression model was established based on the remaining variables after each iteration. The RMSE value was obtained and recorded in each iteration; the value of *p* was updated at the same time, so *p* = r and i = i + 1.

##### Step 5

If i ≤ N, it returned to step 1 for the next iteration; otherwise, it executed step 6.

##### Step 6

The combination of variables corresponding to the minimum RMSE value was selected as the optimal variables.

### 2.6. Performance Evaluation of Methods

The predictive power and the optimal number of LVs of the different models was assessed using Monte Carlo cross-validation (MCCV). Compared with other cross-validation methods, the credibility of model evaluation can be improved by using the random sampling method in the MCCV, and the risk of model over-fitting can be reduced. We selected part of the samples as a calibration set (a total of 145 pears) and used the remaining samples for external verification (a total of 50 pears) using the random method. We repeated the process 100 times and evaluated the performance of the models using the determination coefficient of cross-validation (Rc(*p*)) and RMSE of cross-validation (RMSEcv(*p*)), as follows:

(9) $$R_{c(p)}^{}=\sqrt{1-\frac{\sum_{i-1}^{n}({\hat{y}}_{i}-y_{i}{)}^{2}}{\sum_{i-1}^{n}({\hat{y}}_{i}-y_{m}{)}^{2}}}$$

(10) $${\mathrm{RMSE}}_{\mathrm{CV}(P)}=\sqrt{\frac{1}{N\times n}\sum_{i-1}^{n}({\hat{y}}_{i}-y_{i}{)}^{2}}$$

where ${\hat{y}}_{i}$ is the predicted value of the i-th sample; y_i_ is the measured value of the i-th sample; y_m_ is the average value of sample set; N is the times of repetition, which was equal to 100 in this paper; and n is the total number of samples. In addition, we also calculated the ratio of the standard error in prediction to the standard deviation of the samples, which is the RPD. An RPD > 2.5 meant excellent model predictions, 2.5 ≥ RPD ≥ 2 meant very good, 2 > RPD ≥ 1.8 meant good, 1.8 > RPD ≥ 1.4 meant fair, 1.4 > RPD ≥ 1 meant poor, and RPD < 1 meant very poor [42]. Generally, a good model had higher Rc, Rp, and RPD values and lower RMSECV and RMSEP values, but the difference between RMSECV and RMSEP also was small [43].

## 3. Results and Discussions

### 3.1. Estimation of the SSC, TA, and TI Using Different Spectral Preprocessing Methods

**Figure 4:**
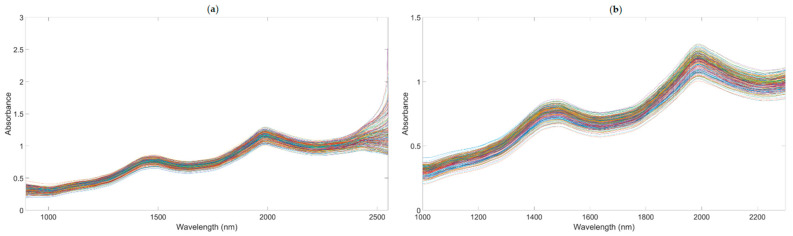
The NIR diffuse reflection spectral of the snow pears. (The lines in figures are the absorbance values of 195 samples at the different wavelengths.). (**a**) NIR raw spectra. (**b**) NIR raw with the range of 1000–2300 nm.

A number of clear absorption peaks were shown in the typical absorbance spectra for the pears within the range of 900 to 2500 nm (Figure 4a). Similar peaks could also be observed at the wavelengths of 1085, 1470, and 1985 nm. From 1000 to 1150 nm, there was a small peak at about 1085 nm, which was related to the third harmonic generation of N-H bonds. After the wavelength at 1300 nm, there was a continuous increase in absorbance; the second and the third peaks appeared at around 1470 and 1985 nm, respectively. The second peak was related to the frequency doubling of C-H, N-H, O-H, and H_2_O bonds. The third peak was related to the combined frequency of H_2_O and O-H bonds, with the strongest absorption band existing at approximately 1985 nm. In general, there was a considerable baseline offset in the 900–2500 nm region. Because of the self-made ultra-compact NIR spectroscopy system, there were many interference spectral signals at the two ends of the spectral line (Figure 4a). Therefore, we obtained the useful wavelength region within the range of 1000 to 2300 nm (Figure 4b).

**Figure 5:**
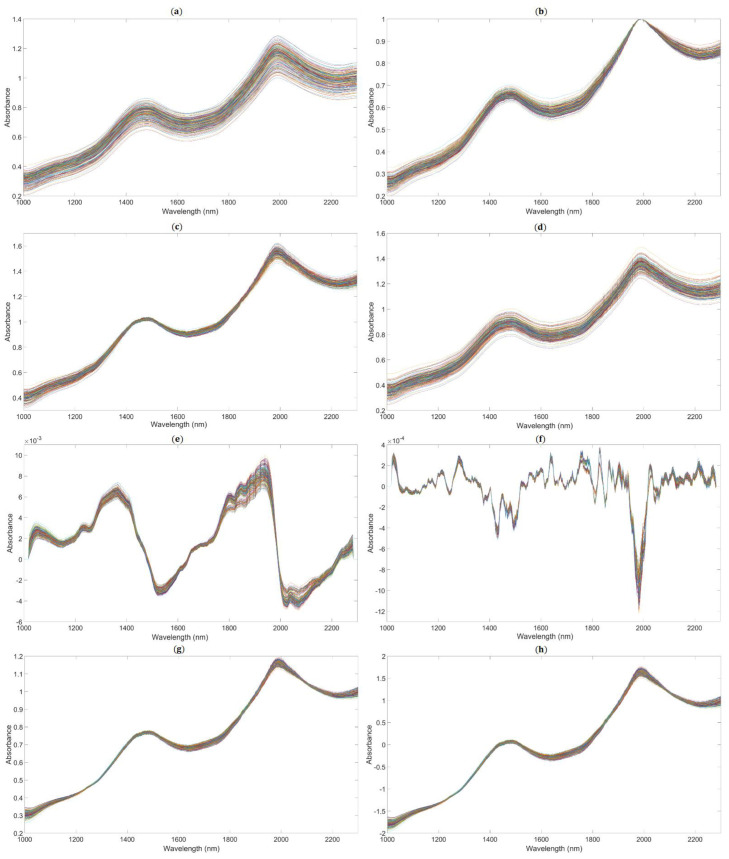
The NIR diffuse reflection spectra preprocessed by different methods. (The lines in figures are the absorbance values of 195 samples after pretreatment at the different wavelengths.). (**a**) Raw spectra by moving average smoothing (MAS). (**b**) Raw spectra by maximum normalization (MaxNorm). (**c**) Raw spectra by mean normalization (MeaNorm). (**d**). Raw spectra by range normalization (RanNorm). (**e**) Raw spectra by Savitzky–Golay first derivative (SG-D1). (**f**) Raw spectra by Savitzky–Golay second derivative (SG-D2). (**g**) Raw spectra by multiplicative scatter correction (MSC). (**h**) Raw spectra by standard normal variate (SNV).

We applied the following methods to get the better performance of the PLSR model based on the full spectrum: spectra preprocessed by MAS (11 points), as shown in Figure 5a; spectra preprocessed by maximum normalization (MaxNorm), as shown in Figure 5b; spectra preprocessed by mean normalization (MeaNorm), as shown in Figure 5c; spectra preprocessed by range normalization (RanNorm), as shown in Figure 5d; spectra preprocessed by SG (11 points) combined with D1, as shown in Figure 5e (SG-D1, Savitzky–Golay first derivative); spectra preprocessed by SG (11 points) combined with D2 (polynomial order = 2, smoothing points = 11), as shown in Figure 5f (SG-D2, Savitzky–Golay second derivative); and spectra preprocessed by MSC (Figure 5g) and SNV (Figure 5h).

The PLSR model for the determination of SSC based on the NIR spectrum, within the range of 900–2500 nm, without any pretreatment (none) is given in Table 2. The result was bad, as the Rc and RMSECV in the calibration set were 0.088 and 0.633%, respectively, and the Rp, RMSEP, and RPD in the prediction set were 0.130, 0.463%, and 1.286, respectively. These results indicated that the spectrum data at two ends of the portable NIR spectrometer produced the noise, and they had adverse effects on the performance for prediction of SSC when using NIR spectroscopy. Therefore, we based subsequent preprocessing and modeling on the NIR spectra in the range of 1000–2300 nm (Raw spectra). For SSC, the MaxNorm, MeaNorm, RanNorm, MSC, and SNV preprocessed spectra-based models were better than that of the Raw spectra. The PLSR model of the Raw spectra was better than those of the MAS, SG-D1, and SG-D2 pretreatment methods. The Rc, Rp, and RPD of the SNV preprocessed spectra-based model for SSC were 0.92, 0.92, and 2.557, respectively, which were greater than those of other pretreatment methods. Therefore, we selected the SNV pretreatment method with a high correlation coefficient and high RPD as the optimal preprocessing model for determination of SSC in snow pears. For TA, the MaxNorm, MeaNorm, RanNorm, SG-D1, MSC, and SNV preprocessed spectra-based models were better than that of the Raw spectra. The PLSR model of the Raw spectra was better than those of the MAS and SG-D2 pretreatment methods. We selected the MSC pretreatment method as the optimal preprocessing model for determination of TA, because the PLSR model based on the NIR spectrum preprocessed by MSC had an Rc, RMSECV, Rp, RMSEP, and RPD of 0.908, 0.142%, 0.906, 0.156%, and 2.356, respectively, and it had a minimal difference between RMSECV and RMSEP, a high correlation coefficient, and a high RPD. For TI, the PLSR model based on the raw NIR spectrum had a good predictive ability with an RPD of 3.692. After the raw NIR spectrum was preprocessed according to the different pretreatment methods, only the MSC and SNV method improved the performance of the PLSR model. The Rc, RMSECV, Rp, RMSEP, and RPD were 0.971, 0.077%, 0.969, 0.089%, and 3.775, respectively. Table 2 shows that the MSC and SNV preprocessed spectra-based models had the best pretreatment effect based on the NIR diffuse reflection spectra for snow pears. The spectrum of the snow pears was affected by uneven particle distribution and different particle size. The MSC and SNV belonged to the scattering correction pretreatment method (see Section 2.4), which eliminated the influence of scattering caused by uneven particle distribution and different particle size on the spectrum.

### 3.2. Estimation of Physicochemical Properties Using the Most Effective Wavelengths

According to previous studies about nondestructive and rapid analysis of physicochemical properties in fruits and vegetables using NIR spectroscopy, variable selection was necessary to select the useful information from NIR spectra. Therefore, NIR spectra had to be extracted by different algorithms after preprocessing to develop the PLSR models.

#### 3.2.1. Estimation of the SSC Property

**Figure 6:**
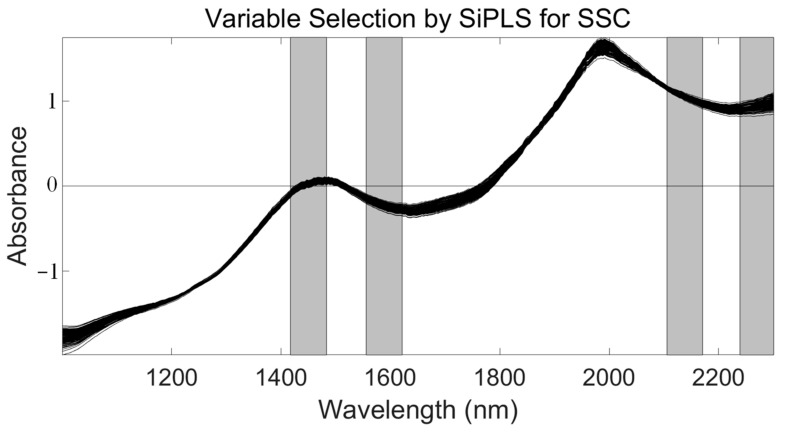
The four synergy subintervals (gray part) selected for SSC by the SiPLS algorithm, based on the SNV preprocessed spectra.

**Figure 7:**
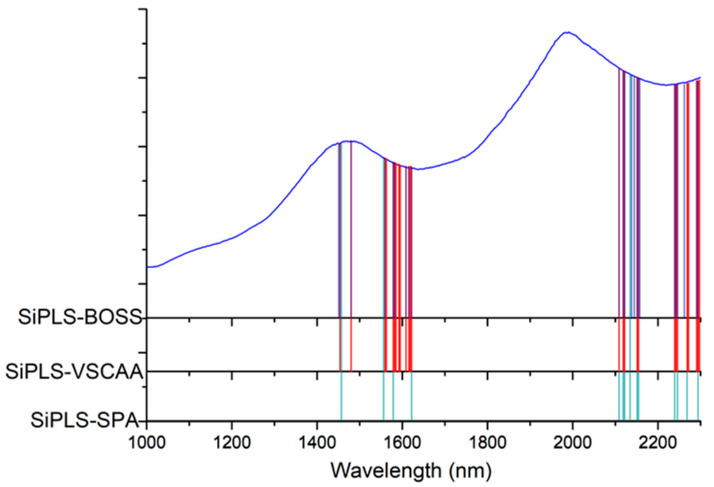
The useful variables selected by SiPLS-SPA, SiPLS-VSCAA, and SiPLS-BOSS. SIPLS-BOSS: synergy interval partial least squares-bootstrapping soft shrinkage; SIPLS-VSCAA: synergy interval partial least squares-variable stability and cluster analysis algorithm; SiPLS-SPA: synergy interval partial least squares-successive projections algorithm.

First, we applied SiPLS (an optimized algorithm of iPLS) to select the effective regions from the NIR spectrum preprocessed by the SNV method. This algorithm combines several different wavelength intervals to combine the wavelength intervals with the largest correlation coefficients and the smallest prediction errors. In this study, we divided the full wavelength into 19 subintervals and selected the 7th, 9th, 17th, and 19th subinterval, as shown in Figure 6. The PLSR model had an Rc, RMSECV, Rp, RMSEP, and RPD of 0.935, 0.209%, 0.925, 0.239%, and 2.654, respectively (Table 3). We used the SPA, VSCAA, and BOSS algorithms to select characteristic wavelengths for the determination of SSC in snow pears. According to different optimization strategies, the useful variables by synergy interval partial least squares-successive projections algorithm (SiPLS-SPA), synergy interval partial least squares-bootstrapping soft shrinkage (SiPLS-BOSS), and synergy interval partial least squares-variable stability and cluster analysis algorithm (SiPLS-VSCAA) totaled 14, 23, and 26, respectively, which could be arranged in the following order: SiPLS-SPA > SiPLS-BOSS > SiPLS-VSCAA (Figure 7 and Table 3).

**Figure 8:**
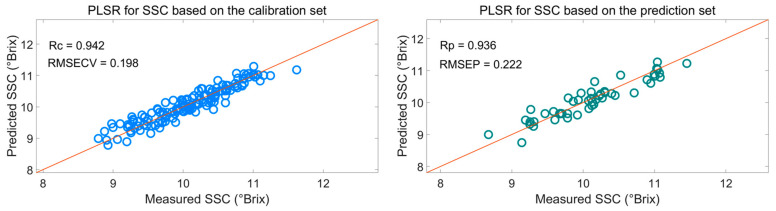
PLSR model for SSC based on the useful wavelengths selected by SiPLS-VSCAA. Rc: correlation coefficient of calibration; RMSECV: root mean square error of cross validation; Rp: correlation coefficient of prediction; RMSEP: root mean square error of prediction.

Figure 7 shows that the effective wavelengths extracted by SiPLS-SPA, SiPLS-BOSS, and SiPLS-VSCAA had similar characteristics. The effective spectra around 1453.49, 1558.87, 1578.87, 1621.35, 2108.81, 2118.58, 2151.13, 2154.39, 2238.93, 2245.42, and 2294.13 nm were simultaneously selected by the three algorithms. This result indicates that the SPA, VSCAA, and BOSS could simplify the PLSR model for determination of SSC in snow pears. For SSC, the absorbance bands between 1453.49 and 1578.87 nm were assigned to the second harmonic generation of hydrogen-containing groups (O-H, C-H), as well as the second harmonic generation of the combined frequency of O-H and C-H. The strong absorption peak of 1621 nm corresponded to the second harmonic generation of C-H. The wavelength from 2108.81 to 2154.39 nm included the combined frequency of O-H and H_2_O, and the 2245.42 and 2294 nm wavelengths carried the information of the combined frequency of C-H, respectively. Finally, we selected the SiPLS-VSCAA as the optimal variable selection algorithm for SSC because of the high coefficient of regression and RPD and the small difference between the RMSECV and RMSEP. The Rc, RMSECV, Rp, RMSEP, and RPD were 0.942, 0.198, 0.936, 0.222, and 2.857, respectively (Figure 8). The number of characteristic wavelengths selected by SiPLS-VSCAA was a little greater than that of SiPLS-SPA and SiPLS-BOSS. The VSCAA not only considered the stability of the regression coefficient of each variable, but it also considered the frequency of the variable. We considered the variable to be useless and removed it only when the stability and frequency of a variable were small at the same time.

**Figure 9:**
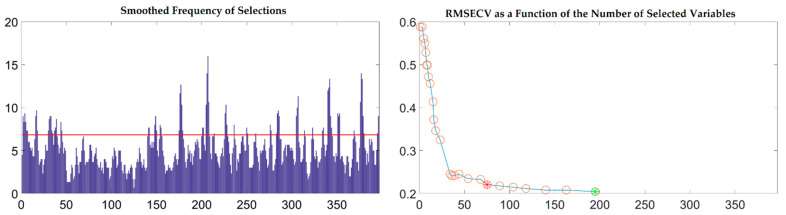
The process of selecting useful wavelengths by using the GA.

**Figure 10:**
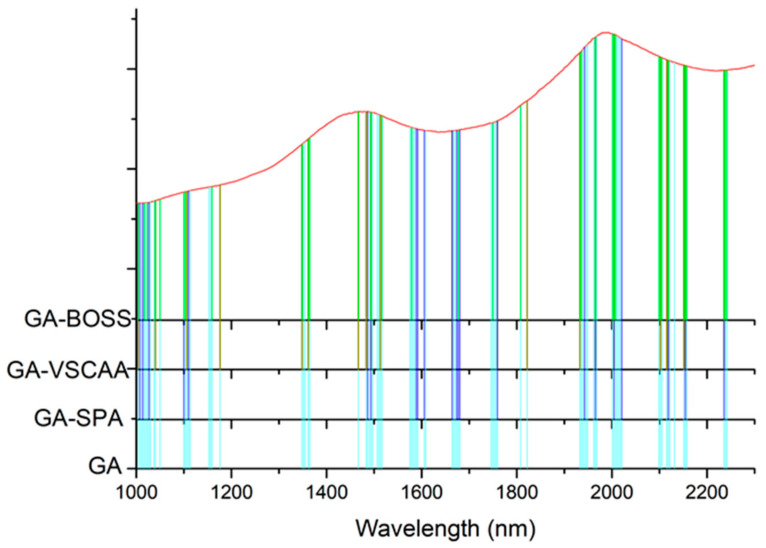
The useful variables selected by GA, GA-SPA, GA-VSCAA, and GA-BOSS. GA-BOSS: genetic algorithm-bootstrapping soft shrinkage; GA-VSCAA: genetic algorithm-variable stability and cluster analysis algorithm; GA-SPA: genetic algorithm-successive projections algorithm; GA: genetic algorithm.

To further reflect the advantages of VSCAA, we applied the GA, GA-SPA, GA-VSCAA, and GA-BOSS to select useful variables to establish the PLSR model for the determination of SSC in snow pears. Figure 9 shows the process of the GA, selecting the characteristic wavelengths. The value of RMSECV was relatively small when the number of selected variables was 86. The number of variables selected by GA and the performance of the PLSR model for SSC were not optimal, however, and the Rc, Rp, and RPD were 0.923, 0.917, and 2.517, respectively. Therefore, we again used the SPA, VSCAA, and BOSS to select wavelengths combined with the GA. The number of selected wavelengths was 22, 22, and 34, as given in Table 4. Figure 10 shows that the distribution of wavelengths selected by the four noted algorithms was not concentrated. The PLS components number of the PLSR model based on the variables selected by GA, GA-SPA, and GA-BOSS were 14,14, and 14, respectively, which were bigger than that of GA-VSCAA (Table 4). The PLSR model based on the variables selected by GA-VSCAA had the best performance in the calibration and prediction sets, as seen in Table 4. Although the variables selected by the GA-VSCAA were better than that of the GA-SPA and GA-BOSS methods, the performance of the PLSR model based on the variables by GA-VSCAA was still not as good as that of the SiPLS-VSCAA. The SiPLS-VSCAA has been proved to be an excellent variable selection combination algorithm to measure the SSC in snow pears, according to the results provided in these figures and tables.

#### 3.2.2. Estimation of TA Property

**Figure 11:**
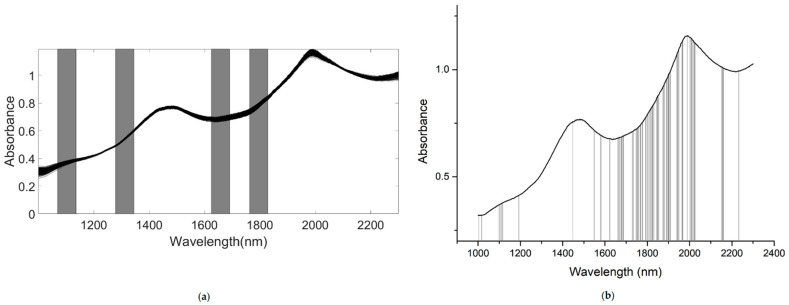
The useful variables selected by the (**a**) SiPLS and (**b**) GA for measuring TA.

**Figure 12:**
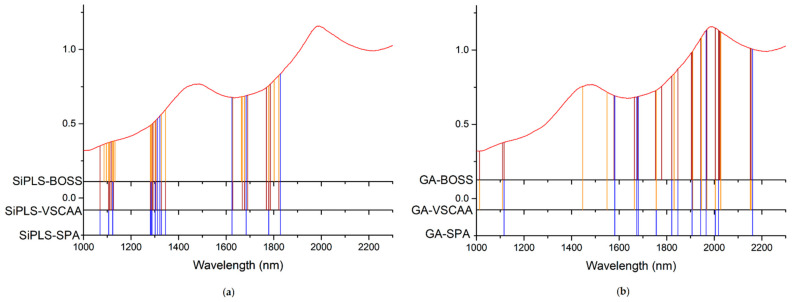
The useful variables selected by the (**a**) SiPLS-SPA, SiPLS-VSCAA, and SiPLS-BOSS and by the (**b**) GA-SPA, GA-VSCAA, and GA-BOSS algorithms for measuring TA.

As for the SSC property measurement, we used SiPLS and GA methods to select the characteristic wavelengths to measure TA, based on the NIR spectrum (first preprocessed by the MSC algorithm) obtained by the NIR spectrometer. The SiPLS method divided the full wavelength into 19 subintervals, and we selected the 2nd, 5th, 10th, and 12th subintervals to establish the PLSR model for TA. The number of useful variables selected by SiPLS was 84, as shown in Figure 11a, and that by the GA was 83, as shown in Figure 11b. The number of variables selected by SiPLS and GA was greater than that by other selection methods, so we combined the SPA, VSCAA, and BOSS algorithms with the SiPLS and GA to further select the more effective wavelengths and build an improved PLSR model for TA. Then, we selected the characteristic variables by the SiPLS-SPA, SiPLS-VSCAA, and SiPLS-BOSS, the numbers of which were 15, 19, and 20, respectively, and by the GA-SPA, GA-VSCAA, and GA-BOSS, the numbers of which were 13, 14, and 20, respectively. These results could be arranged in the order SiPLS-BOSS > SiPLS-VSCAA > SiPLS-SPA and GA-BOSS > GA-VSCAA > GA-SPA (Figure 12 and Table 5).

**Figure 13:**
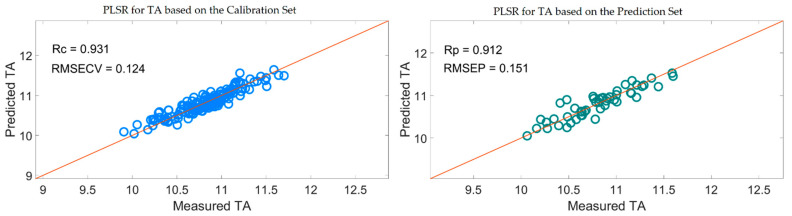
PLSR model for TA based on the useful wavelengths selected by GA-BOSS.

Table 5 shows that the PLSR models for TA based on the useful variables selected by the SiPLS-SPA, SiPLS-VSCAA, and SiPLS-BOSS, as well as by the GA-SPA, GA-VSCAA, and GA-BOSS algorithms, had better prediction ability, with a relatively high Rc, Rp, and RPD and a relatively low RMSECV and RMSEP. These results indicate that these six methods could simplify the PLSR model for determination of TA in snow pears. Figure 12 shows that the distribution of the wavelength selected by SiPLS-SPA, SiPLS-VSCAA, and SiPLS-BOSS was concentrated and that the distribution selected by GA-SPA, GA-VSCAA, and GA-BOSS was relatively dispersed. The effective wavelengths of around 1109.61, 1116.25, 1664.05, 1677.18, 1778.86, and 1821.44 nm were simultaneously selected by the six algorithms. By comparing the PLSR model results for TA based on the variables selected by different algorithms given in Table 5, we determined that the GA-BOSS method was the best variable selection algorithm in this work. The PLSR model for determination of TA in snow pears based on the useful variables selected by the GA-BOSS method had better values of Rc, RMSECV, Rp, RMSEP, and RPD, which were 0.931,0.124%, 0.912, 0.151%, and 2.434 (Figure 13), respectively, and the maximum number of PLS components was 9. The wavelengths selected by the GA-BOSS algorithm were 1013.34, 1109.61, 1116.25, 1578.61, 1664.05, 1677.18, 1752.64, 1778.86, 1821.44, 1847.63, 1903.23, 1906.5, 1942.45, 1968.58, 2004.49, 2017.54, 2020.80, 2024.06, 2151.13, and 2154.39 nm. For TA, the absorbance peak at 1013.34 nm was assigned to the combined frequency and the second harmonic generation of C-H. The absorbance peaks at 1109.61 and 1116.25 nm corresponded to the third harmonic generation of C-H, and the absorbance peaks at 1578.61, 1664.05, and 1677.18 nm corresponded to the second harmonic generation of C-H. The wavelengths at 1903.23, 1906.5, 1942.45, 1968.58, 2004.49, and 2017.54 nm were assigned to the combined frequency of O-H and H_2_O, which affected the prediction ability of the PLSR model. The wavelengths of 2020.80, 2024.06, 2151.13, and 2154.39 nm were assigned to the combined frequency of N-H, which adversely affected the results of the models for measuring TA.

#### 3.2.3. Estimation of TI Property

**Figure 14:**
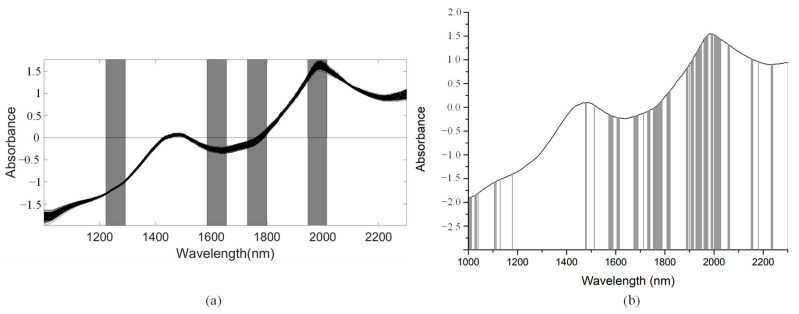
The useful variables selected by the (**a**) SiPLS and (**b**) GA algorithm for measuring TI.

**Figure 15:**
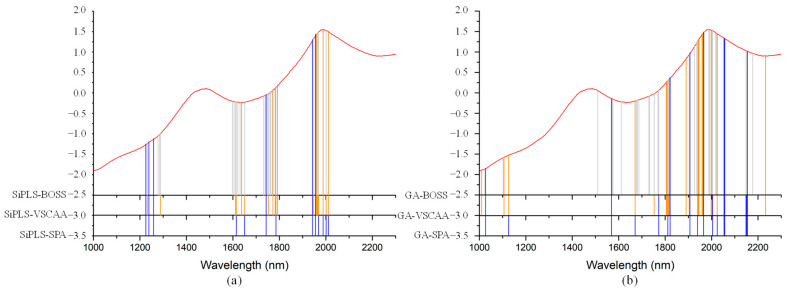
The useful variables selected by the (**a**) SiPLS-SPA, SiPLS-VSCAA, and SiPLS-BOSS and the (**b**) GA-SPA, GA-VSCAA, and GA-BOSS algorithms for measuring TI.

We used the SiPLS algorithm to select characteristic wavelengths for building the PLSR model for determination of TI in snow pears. We divided the full NIR spectrum into 18 subintervals, and we selected the 4th, 9th, 11th, and 14th subinterval as a group dataset for TI. The number of useful variables selected only by SiPLS was 88, as shown in Figure 14a, which was much greater than that by SiPLS-SPA, SiPLS-VSCAA, and SiPLS-BOSS, as shown in Figure 15a. The number of useful variables selected by the three algorithms was 13, 19, and 25, as shown in Table 6. Then, to obtain a better PLSR model for determination of TI in snow pears, we applied GA, GA-SPA, GA-VSCAA, and GA-BOSS methods to extract the useful variables shown in Figure 14b and Figure 15b. Table 6 shows that the number of useful variables selected by the GA algorithm was 100, as it was greater than that by GA-SPA, GA-VSCAA, and GA-BOSS, and the number of selected variables was 18, 23, and 32. For the different optimization strategies, the number of useful variables selected could be arranged in the following order: SiPLS-BOSS > SiPLS-VSCAA > SiPLS-SPA and GA-BOSS > GA-VSCAA > GA-SPA.

**Figure 16:**
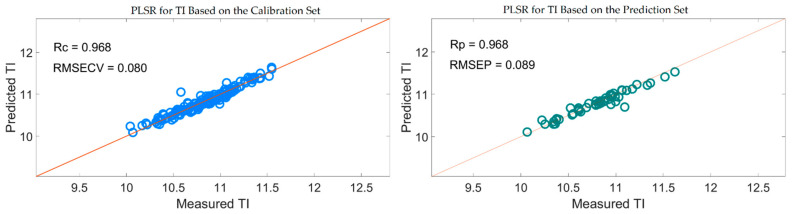
PLSR model for TI based on the useful wavelengths selected by GA-VSCAA.

Figure 15 also shows that the distribution of the wavelengths selected by SiPLS-SPA, SiPLS-VSCAA, and SiPLS-BOSS was concentrated, and that those selected by GA-SPA, GA-VSCAA, and GA-BOSS was relatively dispersed. Figure 15a, b also show that the useful wavelengths around 1614.77, 1755.92, 1775.58, 1948.98, 1965.31, 1971.85, and 2004.49 nm were simultaneously selected by the six algorithms. The wavelengths at 1948.98, 1965.31, 1971.85, and 2004.49 nm were assigned to the combined frequency of O-H and H_2_O, which would have affected the prediction ability of the PLSR model. The fruits had a large number of O-H bands and H_2_O molecule. The maximum number of PLS components based on the variables selected by SiPLS-SPA, SiPLS-VSCAA, and SiPLS-BOSS was 9, 11, and 15, respectively, and that by GA-SPA, GA-VSCAA, and GA-BOSS was 13, 10, and 13, as shown in Table 6. This result indicates that PLSR models based on the variables selected by SiPLS-SPA and GA-VSCAA had relatively smaller PLS components with good performance. The Rc, RMSECV, Rp, RMSEP, and RPD of the PLSR model for determination of TA in snow pears, based on the useful variables selected by the SiPLS-SPA method, were 0.955, 0.095%, 0.957, 0.101%, and 3.326, respectively, and those selected by the GA-VSCAA method were 0.968, 0.080%, 0.968, 0.089%, and 3.775, respectively, as shown in Table 6 and Figure 16. By comparing these results, the PLSR model based on the variables selected by GA-VSCAA had the best prediction ability for TI in pears because of a high correlation coefficient and RPD, minimal difference between RMSECV and RMSEP, and the minimal number of latent variables. The wavelengths selected by the GA-VSCAA algorithm were 1003.38, 1013.34, 1109.61, 1129.51, 1673.9, 1755.92, 1775.58, 1808.34, 1811.62, 1814.89, 1818.17, 1821.44, 1893.42, 1906.5, 1942.45, 1945.72, 1948.98, 1962.05, 1965.31, 2001.22, 2004.49, 2027.32, and 2235.68 nm. For TI, the absorbance peaks at 1003.38 and 1013.34 nm were assigned to the combined frequency and the second harmonic generation of C-H, and the absorbance peaks at 1109.61 and 1129.51 nm corresponded to the third harmonic generation of C-H. The absorbance peaks from 1673.9 nm to 1821.44 nm corresponded to the second harmonic generation of C-H. The wavelengths at 2235.68 nm were assigned to the combined frequency of N-H, which had a bad influence on the measurement model of TI.

## 4. Conclusions

Based on the self-made NIR-spectrum detector, the analysis results showed that the VSCAA proposed in this paper was a good method for selecting effective variables to establish the PLSR model for measuring the SSC content in snow pears. To further show the advantages of VSCAA, we used the GA, GA-SPA, GA-VSCAA, and GA-BOSS algorithms to select useful variables, and we established and compared the PLSR models for detecting SSC content. The VSCAA not only considered the stability of the regression coefficient of each variable, but also considered the frequency of the variable. We considered the variable to be useless and removed it only when the stability and frequency of a variable were small at the same time. In addition, for TA and TI, we built and compared the PLSR models based on the useful variables selected by SiPLS, GA, SPA, VSCAA, and BOSS. The prediction PLSR models, based on the variables selected by GA-BOSS for measuring TA and that by GA-VSCAA for detecting TI, were the best models because of the high correlation coefficient and RPD, the minimal difference between RMSECV and RMSEP, and the minimal number of LVs. In fact, a large number of experiments were carried out at different times, and the number of pear samples was 120, 160, and 195, etc., respectively. The results of calculations were basically the same. We successfully showed that the application of the self-made NIR-spectrum detector, combined with the variable selection algorithms and the PLSR model, was feasible for the rapid and nondestructive analysis of internal qualities (SSC, TA, and TI) in snow pears.

## Figures and Tables

**Table 1:** Statistical results of lignin content in snow pears.

Parameters	No. of Samples	Unit	Range	Mean ± SD	SEL
SSC	195	°Brix	8.04~11.78	9.92 ± 0.78	0.06
TA	195	%	0.0851~0.1480	0.1194 ± 0.0102	0.0007
TI	195	-	68.34~100.82	83.4 ± 6.06	0.43

SSC: soluble solid content; TA: titratable acidity; TI: taste index; SD: Standard deviation; SEL: Standard error of laboratory.

**Table 2:** The performance of the PLSR model based on the NIR spectra range of 1000–2300 nm preprocessed by different methods for determination of SSC, TA, and TI in pears.

WR	NVs	Component	PretreatmentMethod	LVs	Calibration		Prediction		RPD
					R_C_	RMSECV	R_P_	RMSEP	
900–2500 nm	512	SSC	none	6	0.088	0.633	0.130	0.493	1.286
1000–2300 nm	397	SSC	***Raw***	***16***	***0.921***	***0.229***	***0.914***	***0.257***	***2.468***
			MAS	19	0.881	0.280	0.887	0.297	2.135
			MaxNorm	17	0.925	0.224	0.917	0.251	2.527
			MeaNorm	16	0.920	0.230	0.917	0.252	2.517
			RanNorm	17	0.926	0.222	0.920	0.247	2.568
			SG-D1	15	0.910	0.244	0.897	0.287	2.210
			SG-D2	11	0.901	0.256	0.893	0.298	2.128
			MSC	16	0.924	0.225	0.917	0.252	2.517
			***SNV***	***15***	***0.920***	***0.231***	***0.920***	***0.248***	***2.557***
		TA	***Raw***	***14***	***0.908***	***0.142***	***0.903***	***0.159***	***2.311***
			MAS	15	0.901	0.147	0.890	0.169	2.175
			MaxNorm	15	0.906	0.143	0.905	0.156	2.356
			MeaNorm	15	0.906	0.144	0.905	0.157	2.341
			RanNorm	15	0.906	0.144	0.905	0.157	2.341
			SG-D1	11	0.902	0.146	0.906	0.157	2.341
			SG-D2	10	0.842	0.186	0.878	0.176	2.088
			***MSC***	***14***	***0.908***	***0.142***	***0.906***	***0.156***	***2.356***
			SNV	13	0.911	0.140	0.904	0.158	2.326
		TI	***Raw***	***14***	***0.971***	***0.077***	***0.968***	***0.091***	***3.692***
			MAS	20	0.962	0.088	0.960	0.100	3.360
			MaxNorm	15	0.970	0.079	0.967	0.091	3.692
			MeaNorm	15	0.970	0.078	0.966	0.091	3.692
			RanNorm	15	0.970	0.078	0.969	0.088	3.818
			SG-D1	14	0.969	0.079	0.962	0.098	3.428
			SG-D2	12	0.947	0.104	0.940	0.117	2.871
			MSC	14	0.971	0.077	0.969	0.089	3.775
			***SNV***	***14***	***0.971***	***0.077***	***0.969***	***0.089***	***3.775***

WR: Wavelength region; NVs: Number of variables; LVs: Number of latent variables; None: No treatment; Raw: NIR spectra in the range of 1000–2300 nm; Rc: correlation coefficient of calibration; RMSECV: root mean square error of cross validation; Rp: correlation coefficient of prediction; RMSEP: root mean square error of prediction; RPD: residual predictive deviation; MAS: moving average smoothing; MaxNorm: maximum normalization; MeaNorm: mean normalization; RanNorm: range normalization; SG-D1: Savitzky–Golay first derivative; SG-D2: Savitzky–Golay second derivative; MSC: multiplicative scatter correction; SNV: standard normal variate.

**Table 3:** PLSR modeling results for SSC based on variables selected by SiPLS, SiPLS-SPA, SiPLS-VSCAA, and SiPLS-BOSS.

Method	Preprocessing	PLS Components	No. of Variables	Calibration Set		Prediction Set		
				Rc	RMSECV	Rp	RMSEP	RPD
SiPLS	SNV	11	83	0.935	0.209	0.925	0.239	2.654
SiPLS-SPA		9	14	0.936	0.208	0.934	0.231	2.745
**SIPLS-VSCAA**		**10**	**26**	**0.942**	**0.198**	**0.936**	**0.222**	**2.857**
SIPLS-BOSS		12	23	0.948	0.188	0.897	0.281	2.257

SiPLS: synergy interval partial least squares; SiPLS-SPA: synergy interval partial least squares-successive projections algorithm; SIPLS-VSCAA: synergy interval partial least squares-variable stability and cluster analysis algorithm; SIPLS-BOSS: synergy interval partial least squares-bootstrapping soft shrinkage.

**Table 4:** PLSR modeling results for SSC based on variables selected by the GA, GA-SPA, GA-VSCAA and GA-BOSS algorithms.

Method	Preprocessing	PLS Components	No. of Variables	Calibration Set		Prediction Set		
				Rc	RMSECV	Rp	RMSEP	RPD
GA	SNV	14	86	0.923	0.227	0.917	0.252	2.517
GA-SPA		14	22	0.897	0.261	0.911	0.264	2.402
GA-VSCAA		11	22	0.918	0.234	0.918	0.234	2.710
GA-BOSS		14	34	0.936	0.208	0.921	0.245	2.589

GA: genetic algorithm; GA-SPA: genetic algorithm-successive projections algorithm; GA-VSCAA: genetic algorithm-variable stability and cluster analysis algorithm; GA-BOSS: genetic algorithm-bootstrapping soft shrinkage.

**Table 5:** PLSR modeling results for TA based on variables selected by different algorithms.

Method	Preprocessing	PLS Components	No. of Variables	Calibration Set		Prediction Set		
				Rc	RMSECV	Rp	RMSEP	RPD
SiPLS	MSC	11	84	0.905	0.144	0.875	0.180	2.042
SiPLS-SPA		11	15	0.911	0.139	0.884	0.172	2.137
SiPLS-SCSPA		9	19	0.907	0.142	0.901	0.158	2.326
SiPLS-BOSS		10	20	0.924	0.129	0.872	0.179	2.053
GA		11	83	0.922	0.131	0.910	0.152	2.418
GA-SPA		9	13	0.926	0.127	0.908	0.153	2.402
GA-SCSPA		13	14	0.923	0.130	0.894	0.166	2.214
**GA-BOSS**		**9**	**20**	**0.931**	**0.124**	**0.912**	**0.151**	**2.434**

**Table 6:** PLSR modeling results for TI based on variables selected by different algorithms.

Method	Preprocessing	PLS Components	No. of Variables	Calibration Set		Prediction Set		
				Rc	RMSECV	Rp	RMSEP	RPD
**SiPLS**	SNV	12	88	0.959	0.091	0.957	0.101	3.326
SiPLS-SPA		9	13	0.955	0.095	0.946	0.111	3.027
SiPLS-VSCAA		11	19	0.962	0.088	0.961	0.094	3.574
SiPLS-BOSS		15	25	0.968	0.081	0.935	0.121	2.777
GA		13	100	0.971	0.076	0.963	0.095	3.536
GA-SPA		13	18	0.958	0.092	0.943	0.117	2.871
**GA-VSCAA**		**10**	**23**	**0.968**	**0.080**	**0.968**	**0.089**	**3.775**
GA-BOSS		13	32	0.976	0.070	0.961	0.095	3.536

## Data Availability

The data presented in this study are available this article.

## Abbreviations

Soluble Solid Content	SSC	Titratable Acidity	TA
Taste Index	TI	Near Infrared	NIR
Variable Stability and Cluster Analysis Algorithm	VSCAA	Synergy interval Partial Least Squares	SiPLS
Genetic Algorithm	GA	Successive Projections Algorithm	SPA
Bootstrapping Soft Shrinkage	BOSS	Partial Least Square Regression	PLSR
Correlation coefficient of calibration	Rc	Root Mean Square Error of Cross Validation	RMSECV
Correlation coefficient of prediction	Rp	Root Mean Square Error of Prediction	RMSEP
Residual Predictive Deviation	RPD	Monte Carlo-Uninformative Variable Elimination	MC-UVE
Multiple Linear Regression	MLR	Least-Square Support Vector Machine	LS-SVM
Competitive Adaptive Reweighted Sampling	CARS	Relative Humidity	RH
Weighted Bootstrap Sampling	WBS	First Derivative	D1
Association of Official Analytical Chemists	AOAC	Multiplicative Scatter Correction	MSC
Second Derivative	D2	Moving Average Smoothing	MAS
Standard Normal Variate	SNV	Monte Carlo Cross-Validation	MCCV
Savitzky–Golay	SG		
